# Reply to ‘Heterogeneity within AML with *CEBPA* mutations; only *CEBPA* double mutations, but not single *CEBPA* mutations are associated with favorable prognosis’

**DOI:** 10.1038/sj.bjc.6605207

**Published:** 2009-07-21

**Authors:** H-A Hou, L-I Lin, C-Y Chen, H-F Tien

**Affiliations:** 1 Department of Internal Medicine, National Taiwan University Hospital and College of Medicine, Taipei, Taiwan; 2 Department of Clinical Laboratory Sciences and Medical Biotechnology, College of Medicine, National Taiwan University, Taipei, Taiwan

**Sir,**

We read with great interest the recent study by Pabst *et al* (2009) disclosing that there is relevant prognostic heterogeneity within AML patients with *CEBPA* mutations and only *CEBPA* double mutations (*CEBPA*^double-mut^), but not single mutations (*CEBPA*^single-mut^), are associated with favourable prognosis in the AML patients. However, the reason why *CEBPA*^single-mut^ patients have a poorer outcome than *CEBPA*^double-mut^ patients remains unclear and a comprehensive study to evaluate the biological difference between these two groups is still lacking.

In this study, we investigated the prevalence and clinical relevance of *CEBPA*^double-mut^ and *CEBPA*^single-mut^ and their association with other genetic changes in a large cohort of 543 consecutive *de novo* AML patients at the National Taiwan University Hospital (NTUH). This study was approved by the Institutional Review Board of NTUH; written informed consents were obtained from all participants in accordance with the Declaration of Helsinki. *CEBPA* mutations were detected by genomic-DNA PCR and direct sequencing as described earlier (Lin *et al*, 2005). Mutational analyses of *FLT3/ITD*, *FLT3/TKD N-RAS*, *K-RAS*, *NPM1*, *CEBPA*, *KIT*, *AML1* and *MLL/PTD* were carried out as previously described (Hou *et al*, 2008).

Among the 543 AML patients recruited, we identified 71 (13.1%) patients with *CEBPA* mutations, including 47 *CEBPA*^double-mut^ and 24 *CEBPA*^single-mut^. Compared with patients who have *CEBPA*^double-mut^, those with *CEBPA*^single-mut^ had lower incidences to express HLA-DR (65 *vs* 96%, *P*=0.0014), CD7 (44 *vs* 79%, *P*=0.006) and CD15 (35 *vs* 85%, *P*<0.0001), but a higher incidence to express CD56 (35 *vs* 11%, *P*=0.038) on leukemia cells. Apart from this, there were no differences in other clinical parameters including age, sex, haemogram, LDH level, FAB subtype and karyotype between these two groups. No matter *CEBPA*^single-mut^ or *CEBPA*^double-mut^, the mutation disappeared at complete remission in all patients who had paired bone marrow samples for analysis and reappeared at relapse.

Patients with *CEBPA*^single-mut^ had a higher incidence of *NPM1* mutation than those with *CEBPA*^double-mut^ (4/24, 16.7 *vs* 0%, *P*=0.0109). There was also a higher incidence of concurrent mutation of *FLT3/ITD*, *FLT3/TKD*, *AML1/RUNX1* or *MLL/PTD* in *CEBPA*^single-mut^ patients than in *CEBPA*^double-mut^ patients (20.8 *vs* 10.6%, 12.5 *vs* 4.3%, 8.3 *vs* 2.1% and 4.2 *vs* 0%, respectively), but the difference did not reach statistical significance. However, when combined together, simultaneous alteration of any one of these four mutations occurred more frequently in the former group than in the latter (37.5 *vs* 14.9%, *P*=0.039). More intriguingly, all four *CEBPA*^single-mut^ patients with *NPM1* mutation also simultaneously had *FLT3/ITD* (2 patients), *FLT3/TKD* (1 patient), or both (1 patient).

In terms of outcome, *CEBPA*^double-mut^ patients had a higher complete remission rate than *CEBPA*^single-mut^ patients (91 *vs* 56.3%, *P*=0.0051). The patients with *CEBPA*^double-mut^ had a significant longer overall survival (OS) than those with CEBPA^wild^ or *CEBPA*^single-mut^ (median: not reached *vs* 29.8 months and 7.5 months; *P*=0.013 and *P*=0.001, respectively; among 3 groups, *P*=0.007, Figure 1A). The same was also true for disease-free survival (DFS) (median: 59 months *vs* 8 months and 4 months; *P*=0.016 and *P*=0.027, respectively; among 3 groups, *P*=0.037). Among the subgroup of patients with normal karyotype, the differences in OS and DFS between *CEBPA*^double-mut^ and *CEBPA*^single-mut^ patients were still obvious (*P*=0.002 and *P*=0.019, respectively, Figure 1B). The multivariate analysis clearly identified *CEBPA*^double-mut^, but not *CEBPA*^single-mut^ as an independent prognostic factor for OS and DFS (hazard ratio 0.362, 95% CI 0.182–0.721, *P*=0.004 and hazard ratio 0.426, 95% CI 0.263–0.691, *P*=0.001, respectively, Table 1).

From the above findings, we hypothesise that the close association of *CEBPA*^single-mut^ with CD56 expression (Raspadori *et al*, 2001) and other poor-risk genetic alterations, such as *FLT3/ITD*, *FLT3/TKD*, *MLL/PTD* and *AML1/RUNX1*, (Schnittger *et al*, 2000; Harada *et al*, 2004; Whitman *et al*, 2008) may partially explain why *CEBPA*^single-mut^ predisposes to inferior outcome than *CEBPA*^double-mut^. We also observed a trend of shorter OS in *CEBPA*^single-mut^ patients who had concurrent *FLT3/ITD*, *FLT3/TKD*,*MLL/PTD* or *AML1/RUNX1* mutation than those who did not (*P*=0.064, Figure 2).

In summary, about one-third of patients with *CEBPA* mutations had *CEBPA*^single-mut^, which were closely associated with CD56 expression but inversely correlated with HLA-DR, CD7 and CD15 expression. Compared with patients who have *CEBPA*^double-mut^, those with *CEBPA*^single-mut^ had a higher incidence of concurrent *FLT3/ITD*, *FLT3/TKD*, *MLL/PTD* or *AML1/RUNX1* mutation and had a poorer prognosis. This study provides evidences independently from previous ones, stressing the differences in biological characteristics between *CEBPA*^single-mut^ and *CEBPA*^double-mut^ AML and their possible prognostic implication. Further studies are necessary to clarify whether the close association of *CEBPA*^single-mut^ with CD56 expression and other poor-risk gene alterations contributes to the poorer outcome of this group of patients.

## Conflict of interest

The authors declare no conflict of interest.

## Figures and Tables

**Figure 1:**
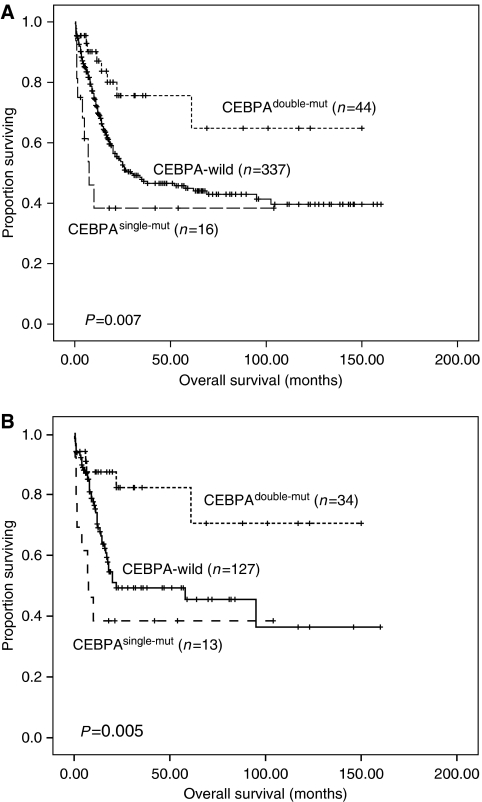
Kaplan–Meier survival curves of overall survival (OS) stratified by different status of *CEBPA* mutation at diagnosis among total patients (**A**) and in the subgroup of patients with normal karyotype (**B**). Only patients receiving standard chemotherapy were enrolled into survival analysis. Among total patients, *P*-value for OS of *CEBPA*^double-mut^ *vs CEBPA*^wild^ patients was 0.013, for *CEBPA*^double-mut^ *vs CEBPA*^single-mut^ patients, 0.001, and among three groups, 0.007 (**A**). In the subgroup of patients with normal karyotype, *P*-value for OS of *CEBPA*^double-mut^ *vs CEBPA*^wild^ patients was 0.01, for *CEBPA*^double-mut^ *vs CEBPA*^single-mut^ patients was 0.002 and among three groups it was 0.005 (**B**).

**Figure 2:**
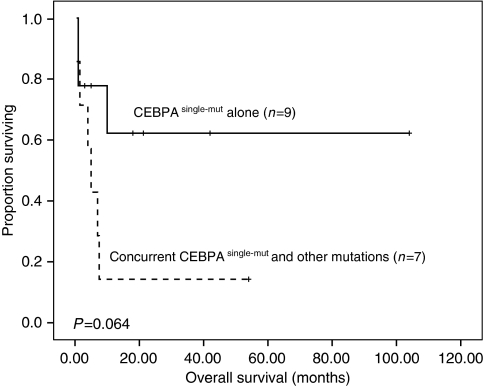
Kaplan–Meier survival curves of overall survival (OS) in the *CEBPA*^single−mut^ patients with and without concurrent *FLT3/ITD*, *FLT3/TKD*, *MLL/PTD* or *AML1/RUNX1* mutation.

**Table 1:** Multivariate analysis for overall and disease-free survival^a^

	**Overall survival**		**Disease-free survival**	
**Variables**	**HR (95% CI)**	***P* value**	**HR (95% CI)**	***P* value**
*CEBPA* ^single-mut^	1.614 (0.743–3.508)	0.227	1.164 (0.630–2.149)	0.629
*CEBPA* ^double-mut^	0.362 (0.182–0.721)	0.004	0.426 (0.263–0.691)	0.001
Karyotype	2.388 (1.774–3.215)	<0.001	2.387 (1.899–3.002)	<0.001
Age^b^	2.741 (1.959–3.836)	<0.001	1.488 (1.137–1.948)	0.004
Sex^c^	0.937 (0.670–1.311)	0.704	1.107 (0.848–1.445)	0.456
WBC^d^	1.524 (1.051–2,209)	0.026	1.396 (1.031–1.890)	0.031
*FLT3/ITD*	1.798 (1.232–2.624)	0.002	1.843 (1.350–2.515)	<0.001
*AML1/RUNX1*	1.755 (1.036–2.972)	0.036	1.410 (0.909–2.187)	0.125
*NPM1*	0.500 (0.317–0.789)	0.003	0.482 (0.332–0.699)	<0.001

Abbreviations: CI=confidence interval; HR=hazard ratio.

a Including 397 patients who received standard chemotherapy. Those patients who did not receive chemotherapy or only low dose chemotherapy were excluded.

b Age greater than 50-years old *vs* less than 50-years old.

c Male *vs* female.

d WBC greater than 50 × 10^9^/l *vs* less than 50 × 10^9^/l.
